# Dementia Prevalence in the CARhES Cohort: Importance of Socioeconomic Level

**DOI:** 10.3390/jcm14207375

**Published:** 2025-10-18

**Authors:** Elena Lobo, Sara Malo, Isabel Aguilar-Palacio, Sara Castel-Feced, Lina Maldonado, Concepción De la Cámara, María José Rabanaque

**Affiliations:** 1 Department of Preventive Medicine and Public Health, Universidad de Zaragoza, 50009 Zaragoza, Spain; smalo@unizar.es (S.M.); iaguilar@unizar.es (I.A.-P.);; 2 Grupo de Investigación en Psiquiatría de Enlace, Aragon Institute for Health Research (IIS Aragón), 50009 Zaragoza, Spain; 3 Centro de Investigación Biomédica en Red de Salud Mental (CIBERSAM), Ministry of Science and Innovation, 28029 Madrid, Spain; 4 Grupo de Investigación en Servicios Sanitarios de Aragón (GRISSA), Aragon Institute for Health Research (IIS Aragón), 50009 Zaragoza, Spainlmguaje@unizar.es (L.M.); 5 Research Network on Chronicity, Primary Care and Health Promotion (RICAPPS), Carlos III Health Institute (ISCIII), 28029 Madrid, Spain; 6 Department of Statistical Methods, Universidad de Zaragoza, 50001 Zaragoza, Spain; 7 Department of Applied Economy, Universidad de Zaragoza, 50001 Zaragoza, Spain; 8 Psychiatry Service, Hospital Clínico Universitario, 50009 Zaragoza, Spain; 9 Department of Medicine and Psychiatry, Universidad de Zaragoza, 50009 Zaragoza, Spain

**Keywords:** dementia, cardiovascular, sex differences, real-world data

## Abstract

**Objectives**: Dementia preventive strategies might benefit from a comprehensive approach that considers the interplay of biological and social factors. The aim of this study was to examine the prevalence of dementia by sex and age in a cohort of individuals with cardiovascular risk factors, and to assess how sociodemographic and clinical factors are associated with this disease. **Methods**: Cross-sectional study was conducted on ≥55-year-old individuals from the CARhES (CArdiovascular Risk factors for HEalth Services research) Spanish cohort. Real-world data on sociodemographic, clinical, and drug information was obtained. Dementia cases were identified by diagnoses and pharmacological treatment. Age- and sex-stratified logistic regression models and sex-stratified CTree analyses were used. **Results**: The prevalence of dementia among the 323,973 individuals in the cohort was 5.2%, 3.4% in men and 6.6% in women, and it increased with age. In both sex groups, stroke and depression were associated with a higher prevalence of dementia for all the age groups, while sex differences were found in the association of the rest of the sociodemographic and clinical variables with dementia. Being older and with lower socioeconomic status were the most predictive factors of dementia prevalence. Stroke was a stronger indicator in men than in women, while hypertension was nearly twice as significant in women. **Conclusions**: The prevalence of dementia in people with cardiovascular risk factors was 5.2%, similar to that of the general population. Besides age, having a lower socioeconomic level was the most important indicator of dementia, which may justify more resources and care for these populations.

## 1. Introduction

Cardiovascular disorders (CVDs) and dementia are global health issues that share common links. CVDs are the leading cause of death globally, and dementia is a neurodegenerative disease affecting millions of people and their families, whose prevalence is rising rapidly, placing significant pressure on health care systems and society [1].

Hypertension, diabetes, and hyperlipidemia are the main cardiovascular risk factors for stroke and myocardial infarction but have been also linked to poor health and complications in several organ systems, including cognitive decline [2,3]. The Lancet Commission has adopted a life-course perspective of dementia and has placed these risk factors specifically in midlife [4]. Lack of research on older ages, due to explicit or implicit exclusion, results in incomplete findings and knowledge [5]. While cardiovascular risk increases with age, it is not clear how its association with dementia varies through aging, nor its different association between sexes. Recently, we have reported a protective effect on cognitive prognosis for individuals older than 75 years with higher systolic blood pressure levels [6]. However, more studies are needed in order to clarify this question and whether sex determines different associations with the risk factors, as dementia figures are higher in women.

Regarding social determinants, proof on the role of socioeconomic level on dementia is still scarce. It has been observed that a lower social class amplifies vulnerability to adverse brain morphologies [7] and that higher levels of deprivation are associated with higher rates of dementia diagnoses in some populations [8]. From a public health perspective, identifying at-risk populations might inform targeted interventions, but also, describing the populations most affected by the disease can help allocate resources more effectively and equitably.

There is currently no data available on the prevalence of people with cardiovascular risk factors living with dementia, or that had explored to what extent the frequency of dementia in this at-risk population is higher than that documented in the general population. At the same time, it is necessary to understand how sex and the age continuum modulate the associations of the identified risk factors with dementia. Furthermore, dementia is influenced by a complex interplay of biological and social factors, which mostly are studied separately. Prevention and care provision strategies might benefit from a more comprehensive approach.

For the first time, this study aimed to comprehensively assess how common dementia is in a group at cardiovascular risk, instead of in the general population, since the studies previously referenced assume that cardiovascular factors increase the risk of this pathology. The objective was to describe the prevalence of dementia by age and sex in adults ≥55 years old from the CARhES cohort, a Spanish cohort of people with cardiovascular risk factors, and to assess how sociodemographic and clinical factors are associated with this disease, while accounting for the joint and potentially interactive effects of these variables on the outcome.

## 2. Materials and Methods

Observational cross-sectional study was conducted among participants of the CARhES (CArdiovascular Risk factors for HEalth Services research) population-based dynamic cohort of individuals aged >15 years, registered as users of the Aragón Health System, with hypertension, diabetes mellitus, and/or dyslipidemia. Aragón is a Spanish region with 1.3 million inhabitants, among which more than 20% are aged >64 years [9]. For the present study, individuals in the CARhES cohort in 2017 who were ≥55 years old were included.

Data was obtained from BIGAN, a platform from the region’s Public Health System for the secondary use of health data that includes quantitative real-world data extracted from the following: Users Database; Pharmaceutical Dispensation Database; Minimum Basic Data Set database; Emergency Database; Primary Care Database; Adjusted Morbidity Groups. More information on the CARhES cohort can be found at Aguilar-Palacio et al. [10].

The protocol of the CARhES cohort was approved by the Clinical Research Ethics Committee of Aragón (CEICA. PI21/148).

### 2.1. Definition of Dementia Cases

Patients with a code of dementia diagnoses in the Minimum Basic Data Set Database (MBDSD) (ICD-10: F00, F01, F02, F03, G30, G31); and/or in the Emergency Database (ED) (ICD-9: 294.1, 394.2, 331.0, 331.1, 331.8); and/or in Adjusted Morbidity Groups (“Demencia”); and/or in Primary Care Database (CIAP-1 updated 2017: P70 or P20 and “Demencia” or “Alzheimer” or “Dem” or “alz” in the label); and/or patients who had a prescription or dispensation of antidementia drugs in the Pharmaceutical Dispensation Database (ATC codes: N06DA and/or N06DX) were included in the analyses.

In order to avoid possible selection bias, patients not identified by the algorithm described and who had a diagnosis of cognitive decline in MBDSD, ED, and CIAP-1 were excluded from the analyses.

### 2.2. Risk Factors for Dementia

Risk factors for dementia included age, as a continuous variable and categorized as 55–79, 80–89, and ≥90 years old, as well as sex, immigrant status (Spanish origin as native), and place of residence (rural/urban) as dichotomous variables. Socioeconomic level, determined by pharmacy copayment level and type of economic activity, was codified with three mutually exclusive categories: (1) employed individuals earning/with a contributory pension of <EUR 18,000 per annum (p.a.); (2) employed individuals earning/individuals with a contributory pension of ≥EUR 18,000 p.a.; (3) others—individuals receiving an unemployment allowance, individuals receiving free medicines, and other situations not included in the aforementioned categories.

Clinical factors comprised the diseases: stroke, heart failure, ischemic heart disease, chronic obstructive pulmonary disease (COPD), chronic kidney disease, cirrhosis, depression, osteoporosis, arthritis, and obesity, and comprised the cardiovascular risk factors: hyperlipidemia, hypertension, and diabetes.

### 2.3. Statistical Analysis

Mean and standard deviation (SD) and the frequency and percentage were used for variables description. We defined age groups based on annual trend changes in dementia prevalence on a logarithmic scale, estimated with the R package v3.5.2. segmented [11]. Chi-squared and T-student tests for categorical and continuous variables were used to compare the differences between people with and without dementia.

Logistic regression analyses were applied in order to provide a well-established statistical framework for estimating associations between sociodemographic and clinical characteristics and dementia prevalence, offering coefficients that are easy to interpret in terms of odds ratios. To study the importance level of sociodemographic and clinical factors in the presence of dementia, Conditional Inference Tree (CTree) analyses were used [12] in order to capture non-linear effects and complex interactions between variables. CTree relies on a formal statistical hypothesis testing framework to select splitting variables, which reduces bias, prevents overfitting, and results in more reliable partitions, producing a transparent decision structure that is easy to visualize, making it particularly suitable when the goal is not only prediction but also understanding the underlying relationships in the data. ROSE balance was applied to the training dataset to address the imbalance in the dependent variable (dementia), ensuring that validation was performed on the test set without altering its distribution. The sample was divided into two subsets: training and test, following a 70–30% ratio. For the final CTree models, multiple parameter tuning experiments were conducted. The optimal configuration was identified as a maximum depth of 8 nodes and a minimum of 300 observations per terminal node. The model was trained on the training subset, evaluated on the test subset, and subsequently applied to the entire population. Variables included in the model where clinical and sociodemographic factors related to the development of dementia: sex, age, socioeconomic level, immigrant status, place of residence, stroke, heart failure, ischemic heart disease, diabetes, hyperlipidemia, hypertension, depression, chronic obstructive pulmonary disease (COPD), and chronic kidney disease. Model performance was validated using metrics such as the Area Under the Curve (AUC) and the F1-Score, ensuring a robust evaluation of its predictive capabilities.

All analyses included stratification by sex, and prevalence and logistic regression analyses were also stratified by age groups. All analyses were performed using the R software v3.5.2.

## 3. Results

After excluding 926 subjects with cognitive decline, a total of 323,973 individuals ≥55 years old from the CARhES cohort were included (Table 1). There were 175,496 (54.2%) women; the mean age was 71.6 years; and most members of the study population were in the lowest socioeconomic level (66.4%), were native (95.8%), and lived in urban areas (69.9%). Table 1 shows the frequency of each clinical feature. The segmented analysis by age identified two tendency change points: 79 and 89 years old.

The prevalence of dementia was 5.2%, with a total of 16,779 cases, being 3.4% (5080) in men and 6.6% (11,699) in women. The prevalence increased with age, passing from 1.7% in the 55–79-years-old group to 13% in 80–89 year olds, and up to 20.3% at 90 or more years old.

As shown in Table 1, compared with individuals without dementia, those with dementia were more frequently women, older, native, with a low socioeconomic level; had more frequently stroke, heart failure, ischemic heart disease, COPD, chronic kidney disease, osteoporosis, hypertension, and diabetes; and less frequently were living in urban areas and had cirrhosis, arthritis, obesity, and hyperlipidemia.

**Figure 1:**
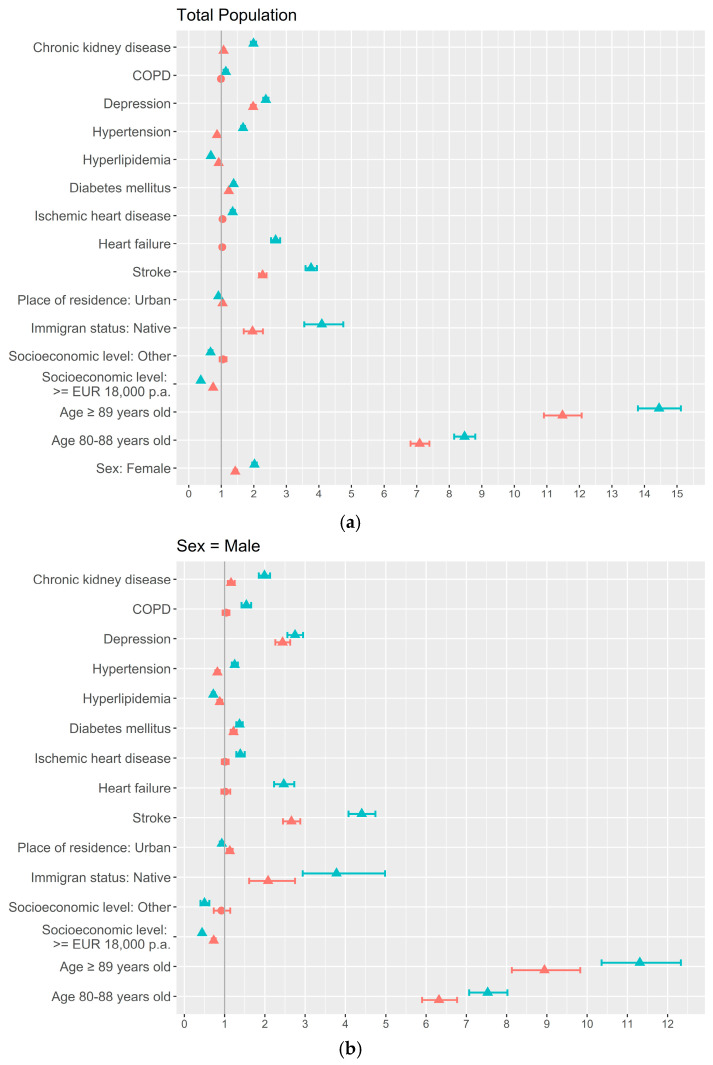

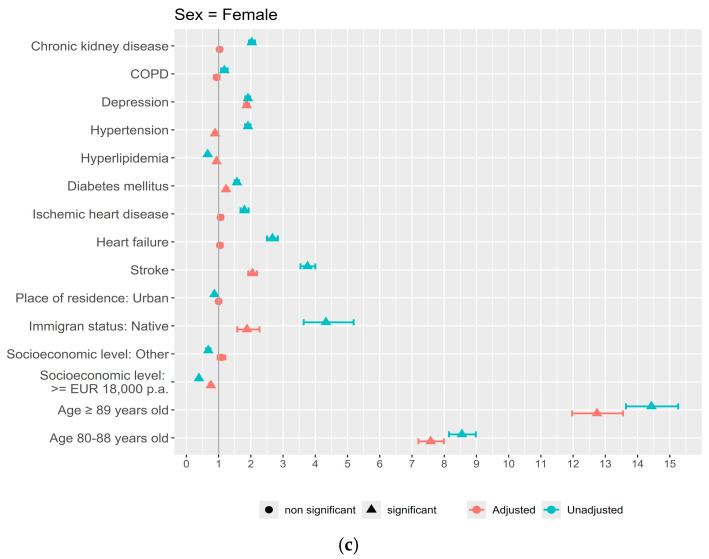
Sociodemographic and clinical factors associated with dementia by sex: uni- and multivariate adjusted analysis using logistic regression: (**a**) Total population; (**b**) Men; (**c**) Women.

**Figure 2:**
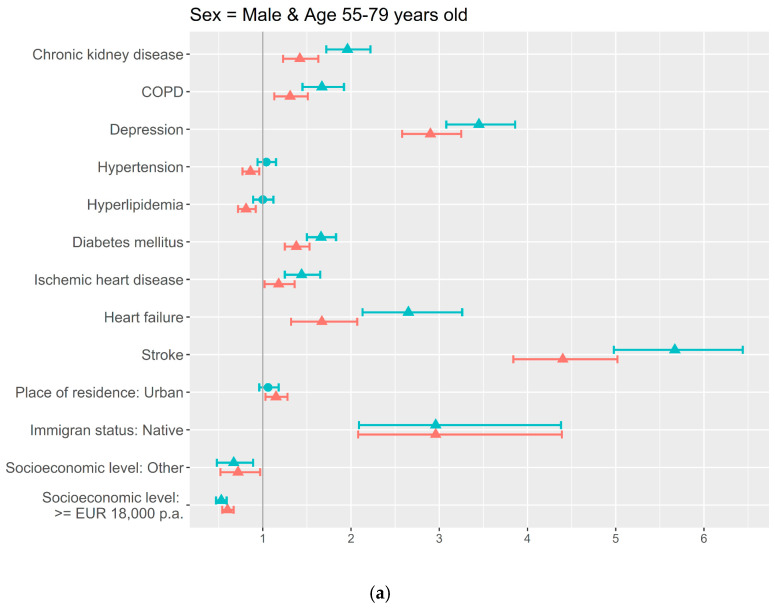

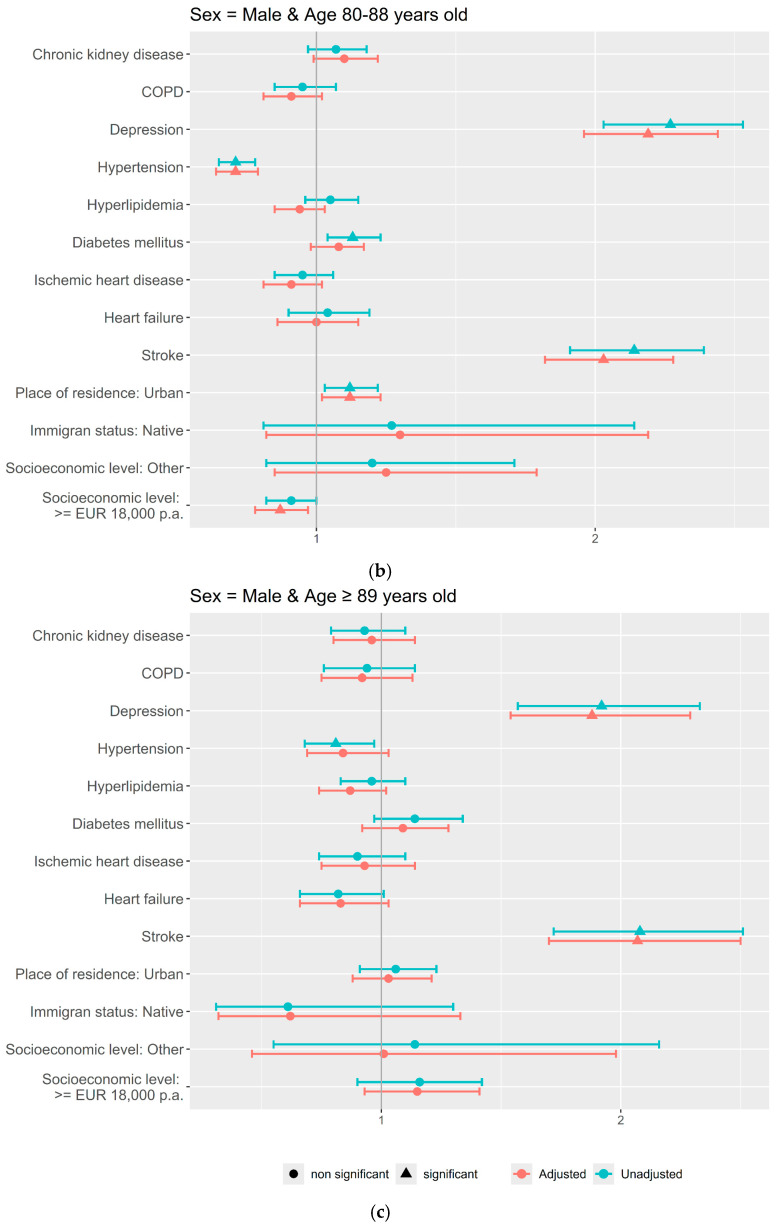
Sociodemographic and clinical factors associated with dementia by age in Men: uni- and multivariate adjusted analysis using logistic regression: (**a**) 55–79 years old; (**b**) 80–89 years old; (**c**) ≥90 years old.

**Figure 3:**
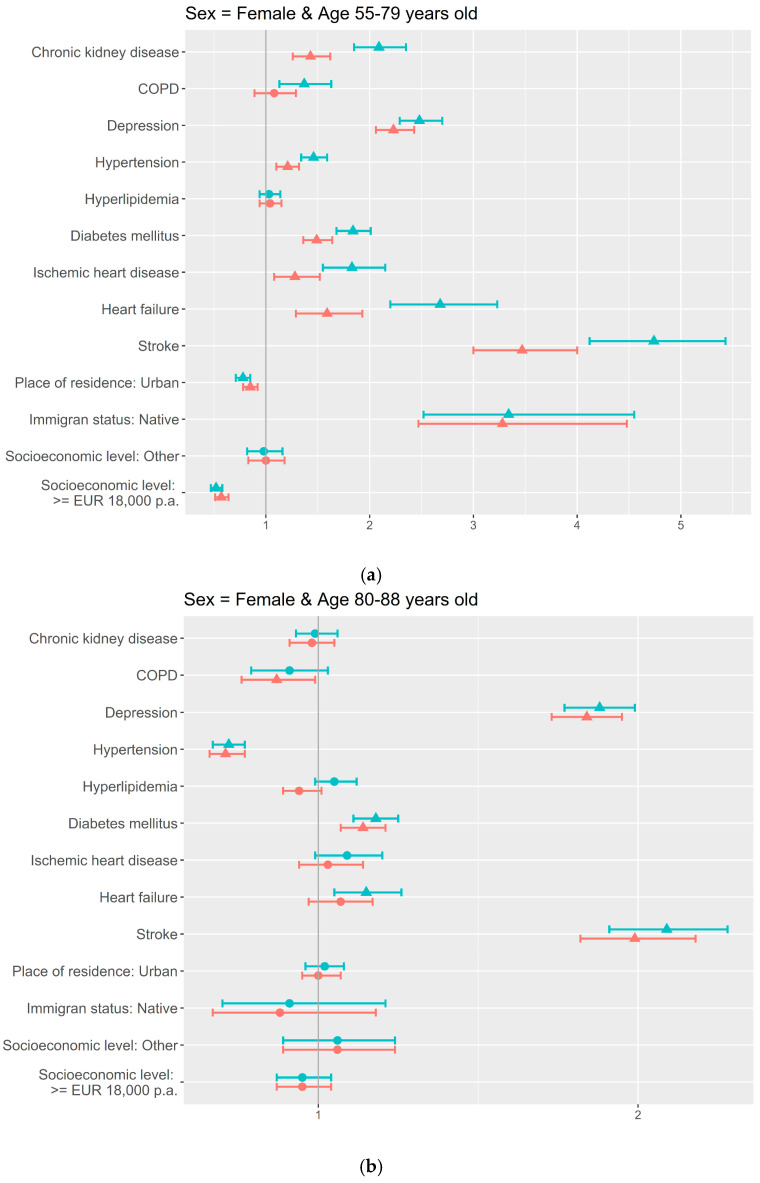

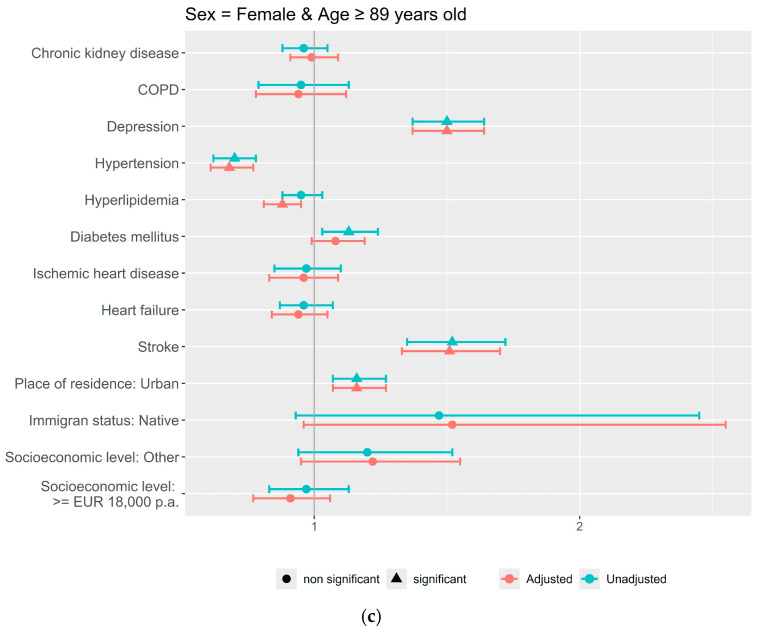
Sociodemographic and clinical factors associated with dementia by age in Women: uni- and multivariate adjusted analysis using logistic regression: (**a**) 55–79 years old; (**b**) 80–89 years old; (**c**) ≥90 years old.

The results of the global and stratified, by sex and age, logistic regression models are shown in Figure 1, Figure 2 and Figure 3. In the adjusted model for the total study population, those factors associated with higher proportions of dementia were feminine sex (OR = 1.4; 95% CI = 1.4–1.5), 80–89 years old (OR = 7.1; 95% CI = 6.8–7.4), native (OR = 2.0; 95% CI = 1.7–2.3), urban residence (OR = 1.0; 95% CI = 1.0–1.1), stroke (OR = 2.3; 95% CI = 2.2–2.4), diabetes (OR = 1.2; 95% CI = 1.2–1.3), depression (OR = 2.0; 95% CI = 1.9–2.1), and chronic kidney disease (OR = 1.1; 95% CI = 1.0–1.1), and the factors associated with a lower frequency of dementia were a higher socioeconomic level (OR = 0.8; 95% CI = 0.7–0.8), hyperlipidemia (OR = 0.9; 95% CI = 0.9–1.0), and hypertension (OR = 0.9; 95% CI = 0.8–0.9) (Figure 1a). Referring to the sex-stratified analyses, similar associations were seen for men and women, with the exception of urban residence and chronic kidney disease that were not associated with the prevalence of dementia in the feminine stratum (Figure 1b,c).

Based on the age thresholds identified, the age- and sex-stratified adjusted models showed more factors associated in the youngest age groups compared to the oldest ones, for both men and women (Figure 2 and Figure 3). In both sex groups, stroke and depression were associated with a higher frequency of dementia for the three age groups; and also in both, men and women, immigrant status, heart failure, and ischemic heart and renal chronic diseases were associated in the 55–79-years-old group.

Sex differences were found for the rest of the sociodemographic and clinical variables. For men, diabetes (OR = 1.4; 95% CI = 1.3–1.5), COPD (OR = 1.3; 95% CI = 1.1–1.5), and urban residence (OR = 1.2; 95% CI = 1.0–1.3) were associated with a higher dementia prevalence in the youngest group, and in the same age group, hypertension (OR = 0.9; 95% CI = 0.8–1.0), hyperlipidemia (OR = 0.8; 95% CI = 0.7–0.9), and the categories of higher socioeconomic level (OR = 0.6; 95% CI = 0.5–0.7) and other (OR = 0.7; 95% CI = 0.5–1.0) were associated with less proportions of dementia (Figure 2a). A higher socioeconomic level (OR = 0.9; 95% CI = 0.8–1.0), urban residence (OR = 1.1; 95% CI = 1–1.2), and hypertension (OR = 0.7; 95% CI = 0.6–0.8) maintained the association in the 80–89-years-old group (Figure 2b).

For women, in the youngest age group, hypertension (OR = 1.2; 95% CI = 1.1–1.3) showed higher proportions of dementia, and, as well as in men, diabetes (OR = 1.5; 95% CI = 1.4–1.6) was also associated, while a higher socioeconomic level (OR = 0.6; 95% CI = 0.5–0.6) and urban residence (OR = 0.9; 95% CI = 0.8–0.9) were inversely associated (Figure 3a). Diabetes maintained the association in the 80–89-years-old group (OR = 1.1; 95% CI = 1.1–1.2) (Figure 3b). Hypertension changed the association to lower proportions of dementia in the two oldest women age groups (OR = 0.7; 95% CI = 0.7–0.8 and OR = 0.7; 95% CI = 0.6–0.8, respectively), as well as urban residence, which in the oldest age group was associated with more dementia (OR = 1.2; 95% CI = 1.1–1.3). COPD (OR = 0.9; 95% CI = 0.8–1.0) and hyperlipidemia (OR = 0.9; 95% CI = 0.8–1.0) showed lower proportions of dementia in the 80–89-years-old and in the oldest women age groups, respectively (Figure 3b,c).

**Figure 4:**
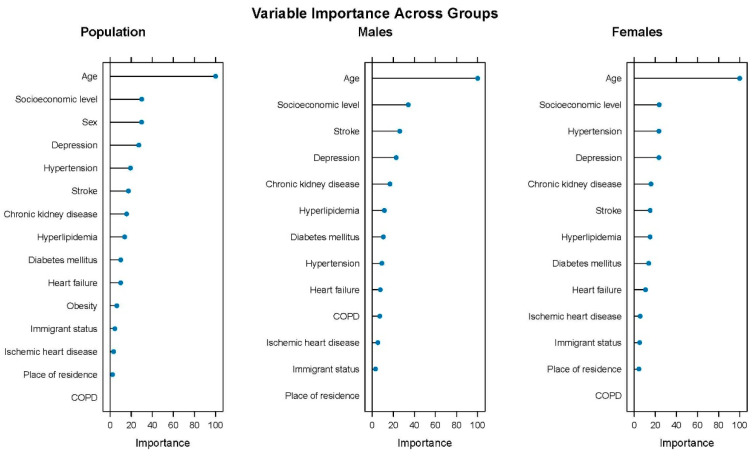
Ranking of dementia-associated factors by overall discriminatory power by sex.

The CTree analyses have ordered the overall discriminatory power of clinical and sociodemographic factors for the presence of dementia (Figure 4). The fit of the model showed a classification capacity (ROC) of 76.4%; the precision was 73.4%, and the F1 score was 23.5%. The Random Forest Plot in Figure 4 shows that the most important factors for explaining the presence of dementia were age and socioeconomic level, globally, and in men and women. Referring to sex differences, while depression was the fourth factor in both, in men, stroke was the third, which together with socioeconomic level and COPD had a greater relative impact compared to in women. Hypertension was the third important factor in women, almost doubling the relative importance compared to in men.

## 4. Discussion

To our knowledge, this is the first study to analyze the epidemiology of dementia in adults ≥55 years old with cardiovascular risk factors. Our results show a prevalence of 5.2%, which is higher in women than in men, 6.6% versus 3.4%, and with a steep rise by age group. Besides age and sex, a cross-sectional association with known modifiable risk factors for dementia such as stroke and depression has been observed. Our findings also indicate sex differences in the association of risk factors with dementia prevalence throughout the age groups. Furthermore, we have found that, for both men and women, the socioeconomic level has shown to be more important than the clinical factors in order to classify individuals based on the presence of dementia.

Remarkably, we cannot claim a higher proportion of dementia cases in this cohort of individuals with cardiovascular risk factors because the prevalence estimates are within the range of those of recent population-based studies, 3.7% in Sweden [13] to 5.9% in Japan [14], 9.1% in China [15], and 10% in the US [16], as well as of the pooled prevalence estimates in the WHO Southeast Asia Region (3%) [17]. Our results are also consistent with the Bacigalupo et al. meta-analysis of the highest quality European studies (7.1%) [18], and with data from geographically closer studies in Madrid (5.9%) [19] and the former ZARADEMP study, in the same region of the present study, which described a global prevalence of 5.9% (women: 7.4%, men: 3.8%) [20]. One possible explanation for this unexpected result might be that the present sample includes 82% of the 65+-years-old population of the region represented [10]. At the same time, the tendency observed of stabilization or declination of the prevalence and incidence rates of dementia in Western countries [21] might contribute to these figures. A survivor bias might be present as well, which could be controlled in future longitudinal studies by documenting incident cases. On the other hand, and referring to comparison with other studies in populations with cardiovascular risk factors, there is only available data coming from people living with diabetes. Although their estimates of the dementia prevalence range from 2.4% [22] to 8.3% [23], they present important methodological differences with our study.

Our findings show that sex and age modulate the association of sociodemographic and clinical characteristics with dementia. Apart from stroke and depression, the jointly sex- and age-stratified analysis illustrated many associations between clinical conditions and dementia at younger ages, which are lost in older ages or even become inversely associated. That is the case of heart failure, ischemic heart disease, diabetes, and chronic kidney disease. One possible explanation could be based on the Protected Survivor Model, which suggests that a minority of the population has a protective factor that lessens the negative impact of a risk factor on cognitive aging. As age increases and due to differential mortality, the proportion of survivors with protection increases [24]. In this research, it is possible that among those surviving to 80 or more years, those protected, albeit presenting risk factors, are numerous and reverse the associations found in the youngest group.

Regarding sex differences, the role of hypertension is intriguing. The analysis with the CTree model shows that the relative importance of hypertension is higher for women than for men, and its association with a higher dementia prevalence was observed only in the youngest women group. This is coherent with the previous literature: a recent review suggests that hypertension, particularly during midlife, increases the risk of late-life dementia more strongly in women than in men, and declining systolic blood pressure in late life is associated with cognitive decline in women but not in men [25]. Women might be more vulnerable than men to the effect of blood pressure [26,27], which could be due to more rapid gray matter atrophy, a higher burden of white matter hyperintensities, and more severe damage to white matter microstructure compared to men [25]. It is also possible that hypertension, jointly with other cardiovascular risk factors, may have had poorer control in women [28]. Anteuffel et al. showed that women were less likely than men to be adherent in their use of chronic medications, and they were less likely to receive the medication treatment and monitoring recommended by clinical guidelines [29]. The age effect noted, especially in women, by which the association of hypertension with higher proportions of dementia in the youngest set changed to an association with a lower prevalence of dementia in the oldest groups, is coherent with previous knowledge. High blood pressure at midlife increases the risk of dementia, while there is still controversy on the adequate blood pressure level at older ages [30]; and, for example, late-life decreases in systolic blood pressure have been associated with cognitive decline, especially in women [31], and cerebrovascular and AD pathology, and mortality [32,33]. In men, we observed lower proportions of dementia for hypertension only in the two youngest groups. An interpretation of this difference between both the male and female strata is that it may be due to sex differences in cardiovascular health that have been previously documented in the literature. Cardiovascular disease develops 7 to 10 years later in women than in men [34], and it might be thought that the relationship between cardiovascular and cognitive health has time differences according to sex.

Although evidence on the interaction between sex and risk factors is increasing, there is still a lack of clear data on whether sex influences the effectiveness of strategies designed to prevent or address these risk factors [35]. From our results, we can consider that the link between hypertension and dementia seems age- and sex-dependent and has implications for clinical practice and public health strategies, as more intense efforts might be needed for the control of hypertension in women before 80 years of age.

Depression is another factor related to dementia that has been identified in terms of importance. Referring to stroke, the higher relative importance for men compared to women needs further exploration, although it might be partially explained by the higher frequency of this condition in men; at the same time, stroke tends to be more severe and deadly in women [36,37]. At the same time, the varying male-to-female prevalence ratios across dementia subtypes, with females being at greater risk for Alzheimer’s disease dementia and males for vascular dementia [35], may partially explain the sex differences observed in our results.

In this study, the most striking finding is that, after age, socioeconomic level was the most important factor for dementia cases classification, above the known risk factors stroke or depression. Several recent European and US cohort studies such as the Survey of Health, Ageing and Retirement in Europe (SHARE), the 3 City Cohort, the English Longitudinal Study of Ageing (ELSA), and the Health and Retirement Study (HRS, US) show that lower socioeconomic status, in terms of education, income, and occupational status, is associated with an increased risk of dementia [38,39,40]. Nevertheless, the underlying mechanisms through which socioeconomic status influences the risk of dementia are still challenging. Deckers et al.’s study concluded that it can be partly explained by modifiable health conditions and lifestyle factors [41], while Letellier et al. showed that cardiovascular health and vascular events had a limited role [39]. From a psychosocial model, Adkins-Jackson et al. explain that limited access to monetary resources decreases opportunities for better quality of life (e.g., education, adequate housing, nutrition, health care, health insurance), which increases the risk of dementia at old age [42]. Education data was not available in this study and might partly explain the association; nevertheless, analogous studies analyzing the causal mediation can provide a better understanding of this finding in order to suggest more equitable interventions. Meanwhile, the observation of a higher prevalence of dementia in lower socioeconomic groups may be important for care facilities and services, as it could justify greater efforts to support these higher-need populations.

Among the strengths of the current study are the population-based cohort in an ageing population in southern Europe that uses real-world data, and the flexible and robust methodologies used for the analysis of the factors associated by Ctree analyses.

There are limitations to be acknowledged. The cross-sectional design hampers assuming causality, and the associations found could be due to reverse causation. For example, in the case of hypertension, dementia itself could have led to poorer management or measurement of this condition, and the observed association may reflect the effects of dementia rather than its true cases. However, our findings may still be relevant in terms of health care provision, as well as they allow us to suggest a hypothesis that can be potentially tested with longitudinal designs. It could be argued that there is a risk of misclassification of dementia cases, as the information is obtained from electronic health databases and may not be fully collected or regularly updated. Dementia cases might have been underestimated, as the formal diagnosis usually takes more time than other diseases, though other studies have provided satisfactory validity measures of similar methodologies and contexts [43,44]. Furthermore, here, we have used several health system databases, representing a more exhaustive procedure. And, while possible disagreement between databases has not been checked, a recent analysis of dementia false positives in health registries in the same country showed they are scarce [43], and possible discordances should only be found between hospital and primary care registries because of different patient profiles with different needs of health care. We cannot ignore the fact that, by study design, hypertension was an inclusion criterion, and the associations found could be an artifact. Nevertheless, hypertension in this sample (66.5%) was similar to other population-based studies showing similar proportions of dementia prevalence [45]. Finally, regarding the CTree analyses, the parameter F1 score might not seem good enough for the model fit; nevertheless, the score is still higher than the prevalence of dementia, which makes it acceptable in this context. Moreover, the precision is close to 80%, indicating that the model produces relatively few false-positive cases; and the rest of the parameters, ROC and precision, were considered adequate.

## 5. Conclusions

As an original contribution, this study has described a prevalence of dementia in people with cardiovascular risk factors of 5.2%, similar to that of the general population, higher in women and with increases through age. A lower socioeconomic level has been identified as the most relevant factor to identify the presence of dementia, after age and the abovementioned clinical factors and diseases, which may justify more resources and care for these populations. Furthermore, our results provide valuable insights into sex-based differences.

## Figures and Tables

**Table 1:** Characteristics of the population by dementia status.

		Population N = 323,973		Individuals without Dementia N = 307,194 (94.8%)		Individuals with Dementia N = 16,779 (5.2%)		*p*-Value *
		Mean	SD	Mean	SD	Mean	SD	
Age		71.6	10.6	70.9	10.3	83.5	7.3	<0.001
		N	%	N	%	N	%	
	55–79 years old	238,193	73.5	234,060	76.2	4133	24.6	<0.001
	80–89 years old	65,434	20.2	56,923	18.5	8511	50.7	
	≥90 years old	20,346	6.3	16,211	5.3	4135	24.6	
	*Missing*	0	0	0	0	0	0	<0.001
Sex	Women	175,496	54.2	163,797	53.3	11,699	69.7	<0.001
	*Missing*	0	0	0	0	0	0	<0.001
Socioeconomic level	<EUR 18,000	215,030	66.4	201,187	65.5	13,843	82.5	<0.001
	≥EUR 18,000	97,664	30.2	95,223	31.0	2441	14.6	
	Other	11,271	3.5	10,777	3.5	494	2.9	
	*Missing*	*8*	*0*	*7*	*0*	*1*	*0.0*	
Immigrant status	Native	310,451	95.8	293,857	95.7	16,594	98.9	<0.001
	*Missing*	*8*	*0*	*7*	*0*	*1*	*0.0*	
Place of residence	Urban	226,427	69.9	215,031	70.0	11,396	67.9	<0.001
	*Missing*	0	0	0	0	0	0	
Stroke		15,312	4.7	12,950	4.2	2362	14.1	<0.001
	*Missing*	1043	0.3	861	0.3	182	1.1	
Heart failure		14,183	4.4	12,492	4.1	1691	10.1	<0.001
	*Missing*	1043	0.3	861	0.3	182	1.1	
Ischemic heart disease		27,464	8.5	25,639	8.4	1825	10.9	<0.001
	*Missing*	1043	0.3	861	0.3	182	1.1	
Depression		54,653	16.9	49,459	16.1	5194	31	<0.001
	*Missing*	1043	0.3	861	0.3	182	1.1	
COPD		23,317	7.2	21,978	7.2	1339	8.0	<0.001
	*Missing*	1043	0.3	861	0.3	182	1.1	
Chronic kidney disease		38,905	12.0	35,476	11.6	3429	20.4	<0.001
	*Missing*	1043	0.3	861	0.3	182	1.1	
Cirrhosis		8877	2.7	8589	2.8	288	1.7	<0.001
	*Missing*	1043	0.3	861	0.3	182	1.1	
Osteoporosis		48,648	15.0	45,051	14.7	3597	21.4	<0.001
	*Missing*	1043	0.3	861	0.3	182	1.1	
Arthritis		8971	2.8	8526	2.8	445	2.7	<0.001
	*Missing*	1043	0.3	861	0.3	182	1.1	
Obesity		50,722	15.7	48,782	15.9	1940	11.6	<0.001
	*Missing*	1043	0.3	861	0.3	182	1.1	
Hyperlipidemia		237,929	73.4	226,911	73.9	11,018	65.7	<0.001
	*Missing*	0	0	0	0	0	0	
Hypertension		215,378	66.5	202,571	65.9	12,807	76.3	<0.001
	*Missing*	0	0	0	0	0	0	
Diabetes		79,214	24.5	74,091	24.1	5123	30.5	<0.001
	*Missing*	0	0	0	0	0	0	

N: number; %: percentage; COPD: chronic obstructive pulmonary disease; *: Chi2 test for categorical variables, T-student for continuous variables.

## Data Availability

Data of this cohort is not freely available due to sensitive information, but we are open to collaborations aligned with the main objectives of the cohort. Researchers interested in conducting data analyses, developing new methodological approaches, or performing cross-national comparisons should contact the Principal Investigators (Isabel Aguilar-Palacio iaguilar@unizar.es and Sara Malo smalo@unizar.es).

## Abbreviations

The following abbreviations are used in this manuscript:

CARhES	CArdiovascular Risk factors for HEalth Services research
CVD	Cardiovascular Disorder
BIGAN	Big Data Sanitario de Aragón
ICD	International Classification of Diseases
MBDSD	Minimum Basic Data Set Database
ED	Emergency Database
CIAP	Primary Care Database
ATC	Anatomical Therapeutic Chemical
